# Overcoming Barriers in Porcine SCNT: A Comprehensive Review of Developmental Challenges and Innovations

**DOI:** 10.3390/ijms27156923

**Published:** 2026-08-01

**Authors:** Xiaoqing Zhou, Jingli Yuan, Shuyi Tan

**Affiliations:** 1 Hainan Provincial Laboratory Animal Center, Sanya Institute, Hainan Academy of Agricultural Sciences, Sanya 572000, China; 2 Institute of Animal Science and Veterinary Medicine, Academy of Agricultural Sciences, Haikou 571100, China

**Keywords:** porcine embryo, SCNT, embryonic development, developmental abnormality, epigenetic reprogramming, mechanistic analysis

## Abstract

Porcine somatic cell nuclear transfer (SCNT) demonstrates significant potential for application in biomedical research, agricultural breeding, and xenotransplantation. However, compared to in vivo fertilized embryos, SCNT embryos exhibit suboptimal developmental capacity, higher rates of abortion, and neonatal abnormalities, which collectively hinder the large-scale application of this technology. Such developmental anomalies arise from a complex interplay of interconnected factors, primarily incomplete epigenetic reprogramming (the core driver), telomere shortening, mitochondrial dysfunction, and technical manipulation-related damage—all of which disrupt the spatiotemporal regulation of gene expression and embryonic lineage commitment. This article provides a critical and integrated review of porcine SCNT research, establishing a coherent conceptual framework that links biological mechanisms, developmental defects, diagnostic tools, and improvement strategies. We emphasize mechanistic insights into key barriers, evaluate the efficacy and limitations of existing diagnostic and therapeutic approaches, and highlight pig-specific biological features that distinguish porcine SCNT from other mammalian models. This review aims to offer a novel perspective on unresolved questions in the field and provide a rigorous reference for in-depth studies on porcine SCNT embryo development.

## 1. Introduction

Porcine somatic cell nuclear transfer (SCNT) technology, recognized as a significant milestone in modern biotechnology and reproductive medicine, has garnered considerable attention from researchers since its emergence. This technology not only offers novel models and tools for biomedical research but also exhibits vast potential in agricultural breeding. Particularly within the domain of xenotransplantation, where pigs are regarded as potential organ donors, the success or failure of porcine SCNT technology directly influences the feasibility and safety of future organ transplants [1,2]. Nevertheless, despite the remarkable progress achieved in porcine SCNT technology, numerous anomalies still manifest during embryonic development, such as inferior developmental capacity, elevated abortion rates, and neonatal abnormalities, which severely constrain the wider application of this technology [3]. Consequently, an in-depth exploration of the underlying causes of abnormal development in porcine SCNT embryos is of significant value for enhancing the success rate and application efficacy of this technology. This review comprehensively surveys porcine SCNT research, from basic principles to embryonic abnormality causes, detection methods, enhancement strategies, and future perspectives, in order to provide a comprehensive reference for advancing research on porcine SCNT embryo development.

## 2. The Biological Basis of Porcine Embryonic Development: Pig-Specific Features and Regulatory Networks

The appropriate progression of early embryonic development depends on the precise and coordinated regulation of gene expression. Studies employing transcriptome analysis of porcine pre-implantation embryos have disclosed the transcriptomic divergence between SCNT-derived and in vivo-derived porcine embryos. For example, a study comparing the transcriptomes of cloned and in vivo embryos from Laiwu and Duroc pigs discovered that, although these embryos presented similar external morphologies during the early developmental stages, they demonstrated significant differences in gene expression profiles. This might account for the lower developmental efficiency of cloned embryos [4]. In-depth single-cell RNA sequencing studies have uncovered transcriptional defects during the reprogramming process of SCNT embryos. Notably, the observed transcriptomic differences between early-cleavage and late-cleavage SCNT embryos imply that early-cleaving embryos may possess greater developmental potential [5,6]. These findings emphasize the influence of incomplete reprogramming in SCNT technology on gene expression.

Blastocyst formation represents the primary lineage commitment event during early embryogenesis in the embryo. The blastocyst consists of the inner cell mass and the trophectoderm: the former develops into the main body of the fetus, while the latter mainly forms the placenta. Key signaling pathways regulating porcine embryonic differentiation, such as the Hippo, WNT, and FGF pathways, have been comprehensively investigated. Recent research utilizing single-cell RNA sequencing technology has analyzed the heterogeneity of porcine blastocyst cells at the single-cell level and identified several species-specific marker genes. For instance, NANOG and SOX2 are utilized to distinguish the inner cell mass, while CDX2 and GATA3 mark the trophectoderm. These findings not only refine the theory of lineage differentiation in pig embryos but also offer potential targets for optimizing in vitro embryo culture systems and enhancing blastocyst quality [7].

In addition to classical transcriptional regulatory mechanisms, epigenetic reprogramming represents another crucial factor throughout early porcine embryonic development. This is specifically reflected in the genome-wide erasure and re-establishment of DNA methylation, as well as dynamic alterations in chromatin accessibility. Relevant studies have indicated that abnormal epigenetic states in porcine SCNT embryos are a major factor contributing to their low cloning efficiency. Histone lysine demethylases KDM5B and KDM5C exhibit high, yet transient, expression levels around the stage of zygotic genome activation (ZGA) in pig embryos, playing a vital role in embryonic development. Inhibition of the mRNA of these demethylases hinders embryo progression to the blastocyst stage and affects the levels of histone modifications such as H3K4me2/3 and H3K9me1, thereby influencing genome activation and stability [8]. These findings emphasize the importance of epigenetic reprogramming in the normal development of porcine SCNT embryos. Aberrant epigenetic regulation can disrupt the normal sequence of gene expression and cell differentiation processes, ultimately leading to the various developmental anomalies observed in cloned embryos.

## 3. The Fundamental Theory of Porcine SCNT: Historical Context and Unresolved Challenges

The development of porcine SCNT has followed a trajectory of technological breakthroughs, but key challenges (e.g., incomplete reprogramming) have persisted, highlighting the need for critical reflection on historical progress. Below, we contextualize major milestones in porcine SCNT, emphasizing how each advance addressed a specific barrier—while identifying gaps that remain unaddressed.

In 1989, Prather et al. initially reported the successful nuclear transfer in porcine embryos. By utilizing unfertilized oocytes as recipients and blastomeres from 4-cell stage embryos as donors, they acquired cloned embryos that developed to the morula/blastocyst stage. After the transfer of these embryos into surrogate sows, viable piglets were successfully produced [9]. As a pioneering endeavor in the realm of porcine nuclear transfer, this study demonstrated the developmental totipotency of porcine blastomere nuclei. Nevertheless, the utilization of embryonic cells as donors possesses limited application value owing to their incapability of replicating specific genotypes.

In 1997, Ian Wilmut’s team declared the birth of “Dolly the sheep,” the world’s first mammal cloned from an adult somatic cell [10]. By employing mammary epithelial cells from an adult sheep as nuclear donors, they demonstrated that even highly differentiated somatic cell nuclei could be reprogrammed to regain totipotency. This achievement was revolutionary, overturning the theory of irreversible cell differentiation and laying the foundation for somatic cell cloning in all large animals, including pigs. Subsequent to the success of Dolly, laboratories across the globe commenced applying this technology to pigs.

By 2000, two research teams nearly simultaneously reported preliminary success in porcine SCNT, utilizing fetal fibroblast cells as donor cells. Polejaeva et al. were the first to report the successful generation of cloned pigs using granulosa cells as donor cells [11]. They adopted a unique “double nuclear transfer” technique, in which the donor nucleus was first transferred into an enucleated oocyte, and following pronucleus formation, it was transferred again into another fertilized and enucleated zygote. Onishi et al. published a more classical approach in the journal Science, where they used fetal fibroblasts as donors and successfully produced the world’s first cloned pigs via conventional SCNT technology through electrofusion and chemical activation [12]. Onishi’s approach, which is still widely used today, marked the maturation of porcine SCNT—but its success rate remained below 5%, highlighting the persistence of developmental barriers.

Subsequent advances integrated gene editing with SCNT: Park et al. (2001) successfully cloned pigs expressing green fluorescent protein using transgenic fetal fibroblast cells [13], and Lai et al. (2002) employed fetal fibroblast cells, in conjunction with gene targeting and SCNT technology, to produce the world’s first α-1,3-galactosyltransferase knockout pigs, achieving a milestone breakthrough in the field of xenotransplantation [14]. In 2014, Hai et al. obtained the first CRISPR gene-edited pig via zygote microinjection and embryo transfer techniques [15]. Although this accomplishment was not based on SCNT technology, it facilitated the application of CRISPR technology in porcine gene editing. Subsequently, research integrating CRISPR technology with SCNT—first performing CRISPR editing in somatic cells followed by nuclear transfer—successfully generated genetically modified pigs [16]. Since then, the integration of CRISPR gene editing technology with SCNT has become the mainstream approach for creating genetically modified pig models. Although the success rate of genetically modified pig models has significantly improved, the issue of abnormal development in cloned pig embryos persists.

## 4. Causes of Abnormal Development in Porcine SCNT Embryos

**Figure 1:**
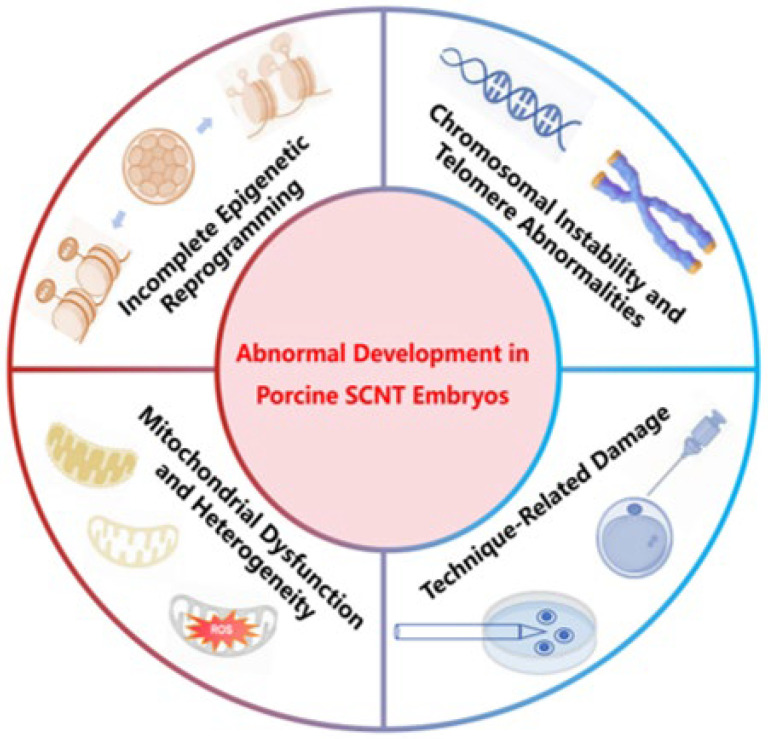
Aberrant development in porcine SCNT embryos: etiology and contributing factors.

The crux of porcine SCNT technology lies in reprogramming highly differentiated somatic cell nuclei back to a totipotent state, a process that emulates the developmental trajectory of naturally fertilized embryos [17]. Nevertheless, in comparison with in vivo-fertilized embryos, SCNT embryos display a notably higher incidence of developmental anomalies. This is predominantly attributed to errors in epigenetic modification during reprogramming, which subsequently result in poor developmental potential, high abortion rates, and neonatal abnormalities [18]. These issues not only limit the efficiency of porcine SCNT technology but also raise concerns about the long-term health and viability of the cloned animals (Figure 1).

### 4.1. Incomplete Epigenetic Reprogramming: The Primary Driver of SCNT Failure

Incomplete epigenetic reprogramming represents a core determinant contributing to the developmental failure of SCNT embryos. Epigenetic modifications can modulate gene expression without altering the DNA sequence. In the event of incomplete reprogramming, it can directly result in the abnormal expression of key genes. Among these, DNA methylation aberrations and histone modification anomalies are the most prevalent [19,20,21,22]. DNA methylation aberrations refer to the inability to completely erase and reconstruct the somatic cell-specific methylation patterns carried by the donor cell nucleus. This leads to the dysregulated expression of imprinted genes, pluripotency-related genes, and developmental genes, ultimately influencing embryonic lineage differentiation and placental development [23,24].

Histone modification anomalies are specifically characterized by reduced levels of histone modifications associated with gene activation (e.g., H3K9ac and H3K4me3) and abnormally elevated levels of modifications linked to gene repression (e.g., H3K9me2/3 and H3K27me3). In particular, H3K9me3 has been verified as one of the major obstacles in porcine SCNT reprogramming, as it impedes transcription factor binding and gene activation [25,26]. Moreover, the histone lysine demethylases KDM5B and KDM5C are expressed during the porcine zygotic genome activation stage. Impairment of their expression can lead to delayed embryonic development, an increase in double-strand breaks, and a reduction in blastocyst formation rates [8].

### 4.2. Mitochondrial Dysfunction and Heterogeneity: Secondary Amplifiers of Epigenetic Defects

Mitochondrial dysfunction is not a primary cause of SCNT failure but rather amplifies the effects of incomplete epigenetic reprogramming by disrupting energy metabolism and redox balance—critical for epigenetic enzyme activity (e.g., DNA methyltransferases, histone demethylases). Porcine SCNT embryos contain mitochondria from both the donor somatic cell and recipient oocyte, leading to mitochondrial DNA (mtDNA) heterogeneity—a phenomenon more pronounced in pigs than in other mammals due to the larger size of porcine somatic cell mitochondria [27,28].

Mechanistically, mtDNA heterogeneity induces a “genetic bottleneck” effect, leading to unequal segregation of donor and oocyte mtDNA during embryonic division. This results in functional incompatibility between nuclear and mitochondrial genomes, disrupting oxidative phosphorylation and reducing ATP production [27]. Reduced ATP levels impair the activity of epigenetic enzymes (e.g., KDM5B/KDM5C), which require ATP for histone demethylation—further exacerbating incomplete reprogramming [8,28]. In porcine SCNT embryos, low expression of mitofusin 1 (a mitochondrial fusion protein) leads to mitochondrial fragmentation, increased reactive oxygen species (ROS) production, and apoptosis—all of which amplify epigenetic defects and reduce developmental potential [27]. Notably, porcine oocytes have lower mtDNA copy numbers than murine or human oocytes, making SCNT embryos more susceptible to mitochondrial dysfunction [28].

### 4.3. Chromosomal Instability and Telomere Abnormalities

Chromosomal instability and telomere abnormalities are secondary consequences of incomplete epigenetic reprogramming, as dysregulated epigenetic marks disrupt mitotic spindle formation and DNA repair mechanisms. Porcine SCNT embryos exhibit higher rates of chromosomal mosaicism (distinct cell populations with different chromosomal compositions) than in vivo embryos—due to errors in mitotic segregation during pre-implantation development [29,30,31,32]. Mechanistically, persistent H3K9me3 marks in SCNT embryos disrupt the expression of genes involved in spindle assembly (e.g., TPX2, AURKA), leading to aneuploidy and chromosomal rearrangements [25].

Telomere shortening is another critical issue in porcine SCNT embryos, as somatic cell telomeres are shorter than embryonic cell telomeres, and the porcine oocyte cytoplasm has limited ability to elongate telomeres [33]. Unlike in cattle, where telomere elongation occurs during SCNT, porcine cloned offspring often have shorter telomeres than in vivo-derived pigs—reducing cellular longevity and increasing the risk of neonatal mortality [33]. This pig-specific telomere defect is exacerbated by incomplete epigenetic reprogramming: dysregulated DNA methylation of telomere-associated genes (e.g., TERT) reduces telomerase activity, further shortening telomeres [33].

Notably, chromosomal instability and telomere abnormalities also contribute to placental dysfunction in porcine SCNT embryos. Porcine cloned placentas often exhibit abnormal vascularization and trophoblast cell proliferation—defects linked to chromosomal mosaicism in TE cells [34]. This creates a vicious cycle: placental dysfunction reduces nutrient and oxygen supply to the embryo, further exacerbating epigenetic and mitochondrial defects.

### 4.4. Technique-Related Damage: Exacerbating Pre-Existing Reprogramming Defects

In SCNT technology, micromanipulation, encompassing oocyte enucleation and somatic nucleus injection or fusion, represents a pivotal step. Throughout this process, oocytes may incur physical damage, consequently influencing their developmental competence [35]. For instance, research has demonstrated that micromanipulation per se can induce endoplasmic reticulum stress. The incorporation of endoplasmic reticulum stress inhibitors (e.g., salubrinal and tauroursodeoxycholic acid, TUDCA) has been shown to enhance the in vitro development of SCNT embryos, indirectly suggesting that micromanipulation may instigate cellular stress and damage [36].

Upon completion of SCNT, the reconstructed embryos necessitate activation treatment to mimic the fertilization process and initiate embryonic development. This procedure is commonly accomplished via electrofusion or chemical activation [37,38]. When sperm penetrates the oocyte, it triggers a series of calcium ion oscillations, which serve as a critical signal for oocyte activation and the initiation of embryonic development. If the intensity or duration of electrofusion or chemical activation is inappropriate, it may fail to effectively replicate this physiological calcium oscillation, resulting in insufficient or excessive activation. Consequently, this can impact pronucleus formation and subsequent embryonic development [39].

## 5. Diagnosis for Abnormal Development in Porcine SCNT Embryos: Critical Evaluation of Methods and Their Limitations

**Figure 2:**
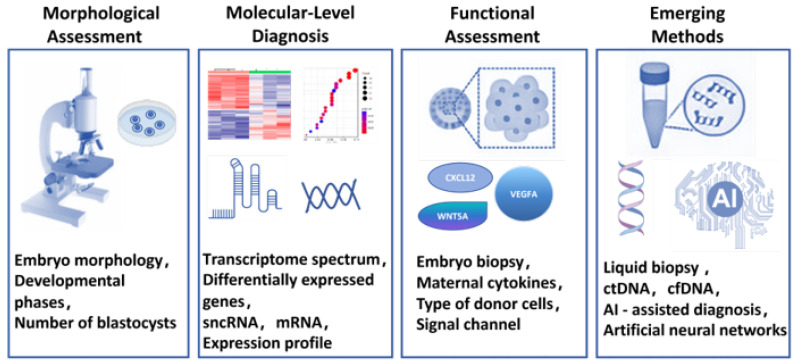
Diagnosis for abnormal development in porcine SCNT embryos.

To tackle the issues of low efficiency and abnormal development in SCNT embryos, researchers are actively investigating diverse diagnostic methods. These methods mainly concentrate on detecting molecular and morphological irregularities during the early stages of embryonic development (Figure 2).

### 5.1. Morphological Assessment: Simple but Insufficient

Morphological assessment (microscopic evaluation of embryo stage, cell number, and blastocyst quality) is the most widely used diagnostic method due to its simplicity and non-invasiveness. However, it has significant limitations: porcine SCNT embryos often exhibit normal morphology at early stages (2-cell, 8-cell) but have underlying molecular defects [30]. For example, early-cleaving SCNT embryos (cleaving before 24 h post-activation) have similar morphology to in vivo embryos but exhibit dysregulated ZGA gene expression—highlighting the lack of correlation between morphology and developmental potential [5].

A critical pig-specific limitation is the difficulty in assessing blastocyst quality: porcine blastocysts have a thicker zona pellucida than other mammals, making it hard to visualize ICM and TE morphology [7]. This reduces the accuracy of morphological assessment for porcine SCNT embryos, as ICM/TE ratio—a key predictor of developmental potential—is often misjudged. Thus, morphological assessment should be used as a preliminary screen, not a definitive diagnostic tool.

### 5.2. Molecular-Level Diagnosis: Powerful but Technically Demanding

Molecular methods (transcriptomics, epigenomics, small RNA profiling) provide mechanistic insights into developmental abnormalities, but their utility is limited by technical complexity and lack of pig-specific reference data. Transcriptomic analysis (e.g., scRNA-seq) can identify dysregulated genes associated with reprogramming failure (e.g., KDM5B, OCT4) [5,24], but requires specialized equipment and bioinformatics expertise. Moreover, most transcriptomic studies use murine or human reference genomes, which fail to capture porcine-specific gene expression patterns [4].

Small non-coding RNA (sncRNA) profiling is a promising pig-specific diagnostic tool: sperm-borne sncRNAs (e.g., miR-34c, miR-125b) are highly expressed in porcine fertilized eggs and enhance SCNT embryo development [40,41]. Reduced expression of these sncRNAs in SCNT embryos correlates with poor developmental potential—providing a specific marker for reprogramming efficiency. However, sncRNA profiling is not widely used due to the lack of standardized protocols for porcine embryos.

### 5.3. Functional Assessment: Invasive but Predictive

Performing embryo biopsy during the early developmental phases (e.g., the 2-cell or 4-cell stage) and monitoring the subsequent developmental trajectory of the biopsied embryos can precisely identify those with enhanced developmental potential, while monitoring the remaining embryo’s development [24]. Maternal cellular factors, including CXCL12, VEGFA, and WNT5A, facilitate porcine oocyte maturation by activating the MAPK pathway and suppressing the canonical WNT pathway, thus conferring developmental competence upon oocytes [42]. Evaluating the expression and activity of these factors can indirectly mirror oocyte quality and its capacity to support the development of SCNT embryos.

Existing research has employed preimplantation genetic testing for aneuploidy (PGT-A) on in vivo-derived porcine embryos and observed high levels of chromosomal abnormalities in the embryos [43]. This finding holds significant theoretical value for elucidating the mechanisms influencing porcine embryo transfer efficiency, suggesting that, in addition to the damage caused by the biopsy procedure itself, the inherently high rate of chromosomal abnormalities in embryos may be a key factor limiting transplantation success. In another study, researchers innovatively combined embryo biopsy techniques with microproteomics to screen for and identify key factors regulating developmental arrest in cloned porcine embryos and validated the biological functions of two candidate regulatory factors, PDCD6 and PLK1, during the development of cloned porcine embryos [44]. Additionally, another study developed a laser-assisted microdissection-based trophectoderm cell biopsy technique for porcine blastocysts, integrating laser technology with the biopsy workflow, thereby providing a new technical approach for sex identification and transcriptome sequencing studies in porcine embryos [45].

### 5.4. Emerging Methods: Promising but Unproven

Emerging diagnostic methods (liquid biopsy, AI-assisted analysis) hold promise for non-invasive, high-throughput diagnosis, but have not been validated for porcine SCNT embryos. Liquid biopsy, which detects cell-free DNA (cfDNA) in embryo culture media, can identify chromosomal abnormalities in human embryos [31], but porcine SCNT embryos release very low levels of cfDNA—making detection challenging. AI-assisted image analysis can automate morphological assessment and integrate multi-omics data [46], but requires large datasets of porcine SCNT embryos to train algorithms—currently lacking in the field.

## 6. Strategies for Improving the Development of Porcine SCNT Embryos: Critical Evaluation and Integrated Approaches

Improving porcine SCNT embryo development requires integrated strategies that target the primary cause (incomplete epigenetic reprogramming) while addressing secondary factors (mitochondrial dysfunction, technical damage). Below, we critically evaluate key improvement strategies, comparing their efficacy, limitations, and pig-specific applicability—replacing the previous descriptive list with a structured, analytical framework (Table 1). We emphasize that the most effective strategies are those that target porcine-specific mechanisms and integrate multiple approaches.

### 6.1. Improving Cellular Reprogramming Efficiency: Targeting the Primary Barrier

**Figure 3:**
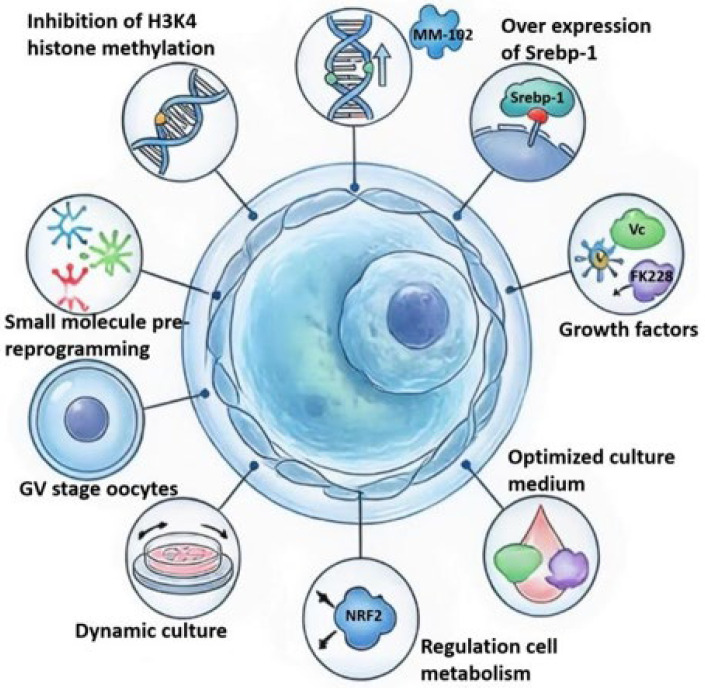
The way to improve cellular reprogramming efficiency.

Enhancing cellular reprogramming efficiency represents a crucial strategy for optimizing the development of porcine SCNT embryos (Figure 3). Aberrant histone methylation has been recognized as a key factor contributing to SCNT failure. Inhibition of H3K4 histone methylation levels (e.g., via the use of MM-102) has been demonstrated to elevate SCNT success rates by reducing H3K4me3 levels [8].

Moreover, investigations have revealed that specific transcription factors and small molecules can augment somatic cell reprogramming efficiency. For example, over-expression of sterol regulatory element-binding protein 1 (Srebp-1) has been shown to promote reprogramming, potentially through interaction with c-Myc to facilitate the expression of pluripotency-related genes [47]. The supplementation of particular growth factors (such as CXCL12, VEGFA, and WNT5A) has been proven to enhance oocyte maturation and activate the MAPK signaling pathway, thereby enhancing reprogramming potential [48].

Prior to nuclear transfer, “pre-reprogramming” donor cells with small molecule cocktails (e.g., vitamin C, FK228) can induce a more open chromatin state, thus improving subsequent reprogramming efficiency within the oocyte cytoplasm [49,50,51]. Research also indicates that utilizing germinal vesicle (GV) stage oocytes as recipients and performing nuclear transfer followed by in vitro maturation may be more favorable for donor nucleus reprogramming, as GV-stage oocytes are enriched with reprogramming-related factors [52].

Furthermore, modification of culture conditions can also exert an influence on cellular reprogramming efficiency. Dynamic culture systems, such as orbital shaking culture, have been shown to significantly enhance reprogramming efficiency. This improvement may be ascribed to the prevention of cell over—confluence and the subsequent up-regulation of p57, thereby facilitating cellular reprogramming [53]. Additionally, metabolic regulation plays a crucial role in reprogramming efficiency. For instance, suppression of NRF2 disrupts the metabolic transition required for successful cellular reprogramming and reduces the formation efficiency of induced pluripotent stem cell (iPSC) colonies, highlighting the critical role of NRF2 in mediating metabolic transitions during cellular reprogramming [54]. Optimization of culture medium components by incorporating natural substances with antioxidant and epigenetic regulatory properties—such as resveratrol and melatonin—can alleviate oxidative stress in SCNT embryos, improve their metabolic status, and indirectly promote reprogramming [28].

### 6.2. Optimizing Donor Cell Selection and Treatment: Reducing Reprogramming Burden

Donor cell selection and treatment are critical for reducing the reprogramming burden, as different cell types have varying epigenetic plasticity. Porcine mesenchymal stem cells (pMSCs) are superior to porcine embryonic fibroblasts (pEFs) as donor cells, as they have lower levels of repressive histone marks (e.g., H3K9me3) and higher epigenetic plasticity—improving blastocyst formation rates by ~28%. Electroporation of reprogramming factors (OCT4, SOX2, KLF4, c-MYC) into pMSCs further enhances their plasticity, but carries the risk of genomic integration—limiting clinical applications [55]. Moreover, pluripotent stem cells are regarded as having higher SCNT efficiency since their epigenetic state is more similar to that of blastomeres, necessitating less reprogramming [56].

Synchronization of donor cells to the G0/G1 phase using roscovitine is another effective strategy for porcine SCNT: roscovitine treatment reduces apoptosis, increases the proportion of G0/G1 cells, and improves full-term development rates by ~23% [57]. Notably, roscovitine is more effective in porcine somatic cells than in murine or bovine cells—highlighting pig-specific sensitivity [57]. Pretreatment of donor cells with the oxidative phosphorylation inhibitor CPI also improves developmental potential by modulating energy metabolism, but high concentrations of CPI are toxic to donor cells [58].

Urine-derived cells are a promising alternative donor cell type for porcine SCNT, as they are non-invasively obtained and have similar developmental potential to pEFs. However, urine-derived cells have higher levels of DNA methylation at pluripotency gene promoters—requiring additional reprogramming strategies (e.g., vitamin C pretreatment) to improve efficiency [59].

### 6.3. Optimizing Recipient Oocytes: Enhancing Reprogramming Capacity

The quality of recipient oocytes is a critical determinant of porcine SCNT efficiency, as oocytes provide the cytoplasmic factors required for reprogramming. In vivo-matured oocytes from multiparous sows are superior to in vitro-matured oocytes or oocytes from gilts, as they have higher levels of reprogramming factors (e.g., KDM5B, CXCL12) and lower levels of repressive epigenetic marks [60,61]. Cloned embryos derived from in vivo-matured oocytes have blastocyst rates ~35% higher than those from in vitro-matured oocytes—making this a highly effective strategy, despite the higher cost of obtaining in vivo-matured oocytes [60,61].

Improving oocyte quality via growth factor supplementation (e.g., FGF2, LIF, IGF1) also enhances SCNT efficiency: supplementation of IVM media with these factors increases blastocyst development rates and birth rates by ~27% [62]. Adding follicular fluid from in vivo-matured follicles to IVM media further improves oocyte quality, as porcine in vivo-matured follicular fluid contains a unique protein profile that supports oocyte maturation [63]. However, follicular fluid composition varies by sow—requiring standardization for consistent results.

### 6.4. Optimizing Nuclear Transfer Procedures: Minimizing Technical Damage

Optimizing the operational procedures of nuclear transfer technology is pivotal for enhancing the developmental efficiency of porcine SCNT embryos. In terms of embryo handling and transfer, research has indicated that factors such as the culture duration of cloned embryos, the ovulatory status of recipients, whether auxiliary embryos are co-transferred, and the site of embryo transplantation all exert an influence on the success rate of pig cloning. For example, transferring cloned embryos that have undergone extended-period culture into pre-ovulatory sows leads to a reduction in pregnancy and farrowing rates, whereas transferring cloned embryos into both fallopian tubes significantly enhances pregnancy rates, farrowing rates, and embryo development rates [64].

Cultivation conditions are of critical importance for embryo survival and developmental competence. Improving the culture environment is regarded as a key strategy for enhancing SCNT efficiency. Implementing low-oxygen culture to regulate the metabolic state of donor cells is an effective optimization approach that can enhance cellular reprogramming capacity and embryo developmental potential [65]. For instance, studies have demonstrated that culturing donor cells under hypoxic conditions significantly improves the early in vivo developmental ability of nuclear-transfer embryos, which is consistent with research on the impact of donor-cell hypoxia on the formation and development of reconstructed embryos in SCNT. Additionally, co-culture systems can optimize the embryonic culture environment. For example, co-culturing bovine oviductal epithelial cells (BOEC) with sperm and oocytes not only facilitates oocyte fertilization but also prevents polyspermy and parthenogenetic activation, thus creating an environment that more closely mimics the in vivo conditions for embryonic development [66].

The enucleation of oocytes can result in mechanical damage to cloned embryos. Chemical agents such as Mitomycin C have been investigated as alternatives to conventional micromanipulation-based enucleation techniques. In bovine SCNT, this approach has shown potential advantages in reducing mechanical damage and improving operational efficiency [67]. The intensity and frequency of electrofusion play a crucial role in the survival and developmental competence of nuclear transfer embryos. Optimizing electrical field parameters can significantly enhance fusion efficiency [17]. Chemical activation reagents (e.g., calcium ionophores) and electroactivation protocols are being comprehensively studied. Combined activation methods have demonstrated a stronger ability to initiate embryonic development, thereby improving embryo quality [68].

## 7. Application of Emerging Technologies in Nuclear Transfer

**Figure 4:**
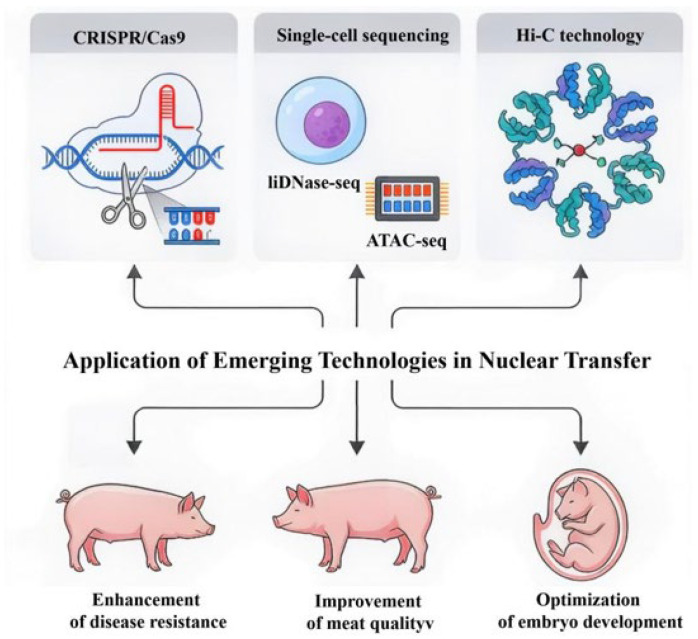
Emerging technologies in nuclear transfer.

The integration of emerging biotechnologies offers novel avenues for optimizing porcine nuclear transfer efficiency (Figure 4). With the continuous progress of CRISPR/Cas9 technology, it has been extensively utilized for precise genome editing in pigs. Through the knockout or knockin of specific genes, it facilitates functional gene research and the breeding of pig breeds with favorable traits, such as enhanced disease resistance and improved meat quality. Meanwhile, the application of single-cell sequencing technologies empowers researchers to comprehensively track gene expression dynamics during porcine embryonic development, uncovering cellular heterogeneity and complexity, thus offering precise theoretical underpinnings for optimizing nuclear transfer techniques.

Currently, several techniques have been adopted to investigate chromatin accessibility in human and mouse pre-implantation embryos to unveil the regulatory principles of open chromatin during embryonic development. These encompass low-input deoxyribonuclease I hypersensitive site sequencing (liDNase-seq) and assay for transposase-accessible chromatin with high-throughput sequencing (ATAC-seq) [69,70,71,72]. Additionally, high-throughput/high-resolution chromosome conformation capture (Hi-C) technology enables a genome-wide exploration of chromatin spatial architecture and reveals dynamic alterations in genome organization [73].

## 8. Interdisciplinary Collaboration Drives Research Progress

Interdisciplinary collaboration plays a pivotal role in research on the development of porcine SCNT embryos. Particularly in the medical field, integration with regenerative medicine can drive the exploration of the application of porcine embryonic stem cells in tissue repair and regeneration. For instance, directing the differentiation of porcine embryonic stem cells into specific cell types and their application in cell replacement therapies for treating human diseases requires the collaborative synergy of multiple disciplines, such as biology, medicine, and materials science [74].

Simultaneously, collaboration with engineering disciplines facilitates the development of more advanced embryo culture systems and operational techniques. For example, 3D printing technology can be employed to construct embryo culture scaffolds that mimic the in vivo environment, providing a more suitable physical microenvironment for embryonic development. In combination with microfluidic technology, precise control over the supply of nutrients and the removal of metabolic waste during embryo culture can be realized, thereby improving embryo development efficiency. Additionally, digital modeling and computer simulation techniques can be used to predict various changes during embryonic development, offering theoretical guidance for experimental design and optimization, and accelerating the progress of porcine SCNT technology.

## 9. Response Concerning Ethical and Social Impacts

**Figure 5:**
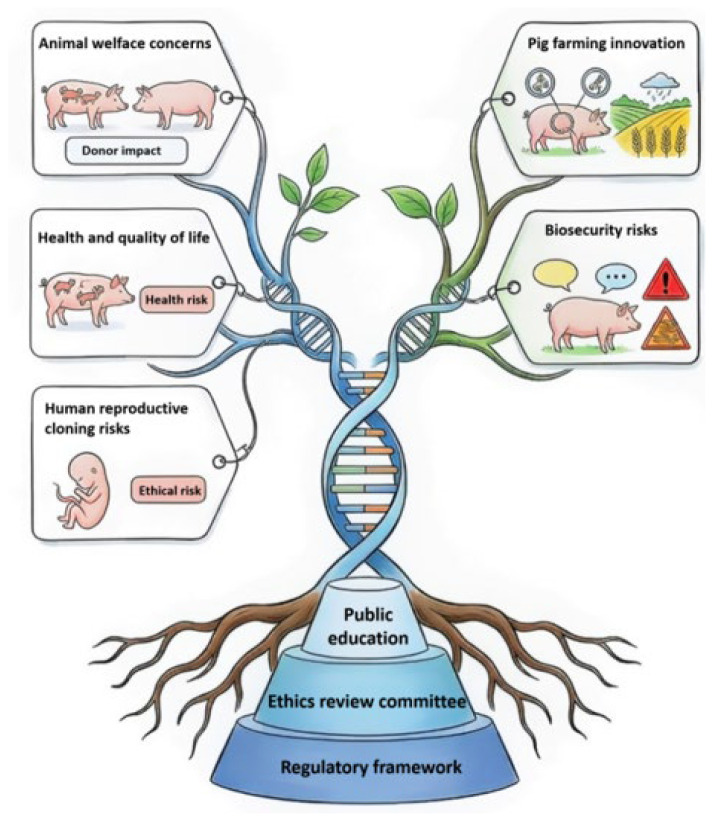
Ethical and social impacts.

Research on porcine SCNT encompasses a series of ethical and social issues (Figure 5). From an ethical perspective, cloning technology may give rise to concerns regarding animal welfare, including the impacts on donor and recipient animals throughout the cloning process, as well as the health and quality of life of cloned animals. Concurrently, when humans apply relevant research findings—for example, using porcine embryonic stem cells for therapeutic applications—it may also spark ethical debates, such as the potential risks of human reproductive cloning and the impacts on human genetic diversity [75].

At the societal level, the application of porcine SCNT technology may have substantial implications for the pig-farming industry. On one hand, this technology can be employed to breed superior breeds, enhance pork production and quality, and satisfy societal demand for meat. On the other hand, if the technology is misused, it could lead to biosecurity issues, such as the ecological impact of genetically modified pigs. Therefore, it is imperative to establish a comprehensive ethical and regulatory framework to ensure the proper utilization of the technology. Simultaneously, public education should be reinforced to enhance awareness and comprehension of the technology, facilitating its healthy and responsible development.

## 10. Conclusions

Porcine SCNT technology holds significant research value and application potential in fields such as animal breeding and biomedicine. However, its development remains constrained by the bottlenecks of low cloning embryo developmental efficiency and high rates of embryonic abnormalities. This paper systematically reviews the causes of embryonic developmental abnormalities in porcine SCNT from three perspectives: donor cell reprogramming, procedural damage during manipulation, and the embryonic microenvironment. It summarizes current diagnostic techniques at the morphological, molecular, and functional levels, while also outlining improvement strategies proposed from aspects such as donor cell treatment and procedural optimization. In recent years, emerging biotechnologies and interdisciplinary research have injected new momentum into this field. The application of technologies such as gene editing and single-cell multi-omics is gradually unraveling the molecular regulatory mechanisms of early porcine embryonic development, offering new possibilities for overcoming the efficiency bottleneck of nuclear transfer. While advancing technological research, we must also confront the ethical and social issues it raises. By improving ethical frameworks, strengthening regulatory systems, and enhancing public communication, we can ensure that porcine SCNT research progresses in a safe, reasonable, and socially beneficial direction. In the future, as our understanding of nuclear reprogramming mechanisms deepens, porcine SCNT technology is bound to play a greater role in related fields.

## Figures and Tables

**Table 1:** Strategies for Improving the Development of Porcine SCNT Embryos.

Strategies	Methods	References
Improving cellular reprogramming efficiency	Inhibition of H3K4 and H3K9 histone methylation, reducing H3K4me3 levels	[8,25,26]
	KDM5B and KDM5C	[8]
	Srebp-1	[47]
	Growth factors: CXCL12, VEGFA, and WNT5A	[48]
	Vitamin C, FK228	[49,50,51]
	Germinal vesicle (GV) stage oocytes	[52]
	Dynamic culture	[53]
	Metabolic regulation: NRF2	[54]
	Optimization of culture medium components	[28]
Optimizing donor cell selection and treatment	pMSCs	[55]
	Transfection of classical reprogramming factors	[55]
	Porcine stem cell	[56]
	Appropriate synchronization of donor cells: roscovitine	[57]
	Pretreatment of donor cells: oxidative phosphorylation inhibitor CPI	[58]
	Porcine urine-derived cells	[59]
	In vivo-matured oocytes	[60,61]
	Oocytes from multiparous sows	[57,58]
	Improving the developmental quality of oocytes: growth factors	[62]
	Adding follicular fluid from in vivo-matured follicles	[63]
Optimizing the operational procedures of nuclear transfer technology	Appropriate culture time of cloned embryos	[64]
	Improving the culture environment: low-oxygen culture	[65]
	Co-culture systems	[66]
	Ameliorate enucleation of oocytes: Mitomycin C	[67]
	Optimizing electrical field parameters	[17]
	Developing combined activation methods	[68]

## Data Availability

No new data were created or analyzed in this study. Data sharing is not applicable to this article.

## References

[1] Yang H.Q., Wu Z.F. (2018). Genome Editing of Pigs for Agriculture and Biomedicine. Front. Genet..

[2] Ryu J., Prather R.S., Lee K. (2018). Use of gene-editing technology to introduce targeted modifications in pigs. J. Anim. Sci. Biotechnol..

[3] Ao Z., Liu D.W., Zhao C.F., Yue Z.M., Shi J.S., Zhou R., Cai G.Y., Zheng E.Q., Li Z.C., Wu Z.F. (2017). Birth weight, umbilical and placental traits in relation to neonatal loss in cloned pigs. Placenta.

[4] He X.Y., Tan C., Li Z.C., Zhao C.F., Shi J.S., Zhou R., Wang X.W., Jiang G.L., Cai G.Y., Liu D.W. (2019). Characterization and comparative analyses of transcriptomes of cloned and in vivo fertilized porcine pre-implantation embryos. Biol. Open.

[5] Liu Z., Xiang G., Xu K., Che J., Xu C., Li K., Wang B., Mu Y. (2020). Transcriptome analyses reveal differential transcriptional profiles in early- and late-dividing porcine somatic cell nuclear transfer embryos. Genes.

[6] Liu Y., Wu F., Zhang L., Wu X., Li D., Xin J., Xie J., Kong F., Wang W., Wu Q. (2018). Transcriptional defects and reprogramming barriers in somatic cell nuclear reprogramming as revealed by single-embryo RNA sequencing. BMC Genom..

[7] Ramos-Ibeas P., Sang F., Zhu Q., Alberio R. (2019). Pluripotency and X chromosome dynamics revealed in pig pre-gastrulating embryos by single cell analysis. Nat. Commun..

[8] Glanzner W.G., Gutierrez K., Rissi V.B., de Macedo M.P., Lopez R., Currin L., Dicks N., Baldassarre H., Agellon L.B., Bordignon V. (2020). Histone lysine demethylases KDM5B and KDM5C modulate genome activation and stability in porcine embryos. Front. Cell Dev. Biol..

[9] Prather R.S., Sims M.M., First N.L. (1989). Nuclear transplantation in early pig embryos. Biol. Reprod..

[10] Wilmut I., Schnieke A.E., McWhir J., Kind A.J., Campbell K.H.S. (1997). Viable offspring derived from fetal and adult mammalian cells. Nature.

[11] Polejaeva I.A., Chen S.H., Vaught T.D., Page R.L., Mullins J., Ball S., Dai Y., Boone J., Walker S., Ayares D.L. (2000). Cloned pigs produced by nuclear transfer from adult somatic cells. Nature.

[12] Onishi A., Iwamoto M., Akita T., Mikawa S., Takeda K., Awata T., Hanada H., Perry A.C. (2000). Pig cloning by microinjection of fetal fibroblast nuclei. Science.

[13] Park K.-W., Cheong H.-T., Lai L., Im G.-S., Kühholzer B., Bonk A., Samuel M., Rieke A., Day B.N., Murphy C.N. (2001). Production of nuclear transfer-derived swine that express the enhanced green fluorescent protein. Anim. Biotechnol..

[14] Lai L., Kolber-Simonds D., Park K.W., Cheong H.T., Greenstein J.L., Im G.S., Samuel M., Bonk A., Rieke A., Day B.N. (2002). Production of α-1,3-galactosyltransferase knockout pigs by nuclear transfer cloning. Science.

[15] Hai T., Teng F., Guo R., Li W., Zhou Q. (2014). One-step generation of knockout pigs by zygote injection of CRISPR/Cas system. Cell Res..

[16] Zhou X., Xin J., Fan N., Zou Q., Huang J., Ouyang Z., Zhao Y., Zhao B., Liu Z., Lai S. (2015). Generation of CRISPR/Cas9-mediated gene-targeted pigs via somatic cell nuclear transfer. Cell. Mol. Life Sci..

[17] Srirattana K., Kaneda M., Parnpai R. (2022). Strategies to improve the efficiency of somatic cell nuclear transfer. Int. J. Mol. Sci..

[18] Loi P., Iuso D., Czernik M., Ogura A. (2016). A new, dynamic era for somatic cell nuclear transfer?. Trends Biotechnol..

[19] Li S., Du W., Li N. (2004). Epigenetic reprogramming in mammalian nuclear transfer. Chin. Sci. Bull..

[20] Ogura A., Matoba S., Inoue K. (2021). 25th anniversary of cloning by somatic-cell nuclear transfer: Epigenetic abnormalities associated with somatic cell nuclear transfer. Reproduction.

[21] Rideout W.M., Eggan K., Jaenisch R. (2001). Nuclear cloning and epigenetic reprogramming of the genome. Science.

[22] Jaenisch R., Bird A. (2003). Epigenetic regulation of gene expression: How the genome integrates intrinsic and environmental signals. Nat. Genet..

[23] Ikeda S. (2023). Current status of genome-wide epigenetic profiling of mammalian preimplantation embryos. Reprod. Med. Biol..

[24] Liu W., Liu X., Wang C., Gao Y., Gao R., Zhao X., Zhao Y., Li J., Wu Y., Xiu W. (2016). Identification of key factors conquering developmental arrest of somatic cell cloned embryos by combining embryo biopsy and single-cell sequencing. Cell Discov..

[25] Matoba S., Liu A., Lu F., Iwabuchi K., Shen L., Inoue A., Zhang R. (2014). Embryonic development following somatic cell nuclear transfer impeded by persisting histone methylation. Cell.

[26] Zhang Z., Zhai Y., Ma X., Zhang S., An X., Yu H., Li Z. (2018). Down-regulation of H3K4me3 by MM-102 facilitates epigenetic reprogramming of porcine somatic cell nuclear transfer embryos. Cell Physiol. Biochem..

[27] Park M.R., Hwang I.S., Kwak T.U., Lim J.H., Hwang S., Cho S.K. (2020). Low expression of mitofusin 1 is associated with mitochondrial dysfunction and apoptosis in porcine somatic cell nuclear transfer embryos. Anim. Sci. J..

[28] Liang S., Jin Y.X., Yuan B., Zhang J.B., Kim N.H. (2017). Melatonin enhances the developmental competence of porcine somatic cell nuclear transfer embryos by preventing DNA damage induced by oxidative stress. Sci. Rep..

[29] Charalambous C., Webster A., Schuh M. (2023). Aneuploidy in mammalian oocytes and the impact of maternal ageing. Nat. Rev. Mol. Cell Biol..

[30] Budrewicz J., Chavez S.L. (2024). Insights into embryonic chromosomal instability: Mechanisms of DNA elimination during mammalian preimplantation development. Front. Cell Dev. Biol..

[31] Hallermayr A., Keßler T., Steinke-Lange V., Heitzer E., Holinski-Feder E., Speicher M. (2023). The utility of liquid biopsy in clinical genetic diagnosis of cancer and monogenic mosaic disorders. Med. Genet..

[32] Ross P.J., Goissis M.D., Martins J.P.N., Chitwood J.L., Pursley J.R., Rosa G.J.M., Cibelli J.B. (2023). Blastocyst cell number and allocation affect the developmental potential and transcriptome of bovine somatic cell nuclear transfer embryos. Stem Cells Dev..

[33] Jeon H.Y., Hyun S.H., Lee G.S., Kim H.S., Kim S., Jeong Y.W., Kang S.K., Lee B.C., Han J.Y., Ahn C. (2005). The analysis of telomere length and telomerase activity in cloned pigs and cows. Mol. Reprod. Dev..

[34] Kim E., Cai L., Choi H., Kim M., Hyun S.H. (2024). Distinct properties of putative trophoblast stem cells established from somatic cell nuclear-transferred pig blastocysts. Biol. Res..

[35] Yoo J.G., Demers S.P., Lian L., Smith L.C. (2007). Developmental arrest and cytoskeletal anomalies of rat embryos reconstructed by somatic cell nuclear transfer. Cloning Stem Cells.

[36] Park Y.R., Park H.B., Kim M.J., Jung B.D., Lee S., Park C.K., Cheong H.T. (2019). Effects of endoplasmic reticulum stress inhibitor treatment during the micromanipulation of somatic cell nuclear transfer in porcine oocytes. Dev. Reprod..

[37] Sugawara A., Sugimura S., Hoshino Y., Sato E. (2009). Development and spindle formation in rat somatic cell nuclear transfer (SCNT) embryos in vitro using porcine recipient oocytes. Zygote.

[38] Han Z., Liang C.G., Cheng Y., Duan X., Zhong Z., Potireddy S., Moncada C., Merali S., Latham K.E. (2010). Oocyte spindle proteomics analysis leading to rescue of chromosome congression defects in cloned embryos. J. Proteome Res..

[39] Keefer C.L. (2015). Artificial cloning of domestic animals. Proc. Natl. Acad. Sci. USA.

[40] Cuthbert J.M. (2020). Comparative Analysis of Small Non-Coding RNA and Messenger RNA Expression in Somatic Cell Nuclear Transfer and In Vitro-Fertilized Bovine Embryos During Early Development Through the Maternal-to-Embryonic Transition. Doctoral Dissertation.

[41] Qin H., Qu P., Hu H., Cao W., Liu H., Zhang Y., Zhao J., Nazira F., Liu E. (2021). Sperm-borne small RNAs improve the developmental competence of pre-implantation cloned embryos in rabbit. Zygote.

[42] Liu X., Hao Y., Li Z., Zhou J., Zhu H., Bu G., Liu Z., Hou X., Zhang X., Miao Y.L. (2020). Maternal cytokines CXCL12, VEGFA, and WNT5A promote porcine oocyte maturation via MAPK activation and canonical WNT inhibition. Front. Cell Dev. Biol..

[43] Jochems R., Canedo-Ribeiro C., Silvestri G., Derks M.F.L., Hamland H., Zak L.J., Knol E.F., Handyside A.H., Grindflek E., Griffin D.K. (2023). Preimplantation Genetic Testing for Aneuploidy (PGT-A) Reveals High Levels of Chromosomal Errors in In Vivo-Derived Pig Embryos, with an Increased Incidence When Produced In Vitro. Cells.

[44] Zhang Y., Yang L., Zhang Y., Liang Y., Zhao H., Li Y., Cai G., Wu Z., Li Z. (2022). Identification of Important Factors Causing Developmental Arrest in Cloned Pig Embryos by Embryo Biopsy Combined with Microproteomics. Int. J. Mol. Sci..

[45] Liu Y., Li Y., Li W., Ji M., Zhang Y., Ma X., Liu Z., Weng X. (2025). Biopsy of Porcine Blastocysts for Sex Identification and Transcriptome Sequencing Based on Laser-Assisted Microdissection. Reprod. Domest. Anim..

[46] Lugtu E.J., Ramos D.B., Agpalza A.J., Cabral E.A., Carandang R.P., Dee J.E., Martinez A., Jose J.E., Santillan A., Bangaoil R. (2022). Artificial neural network in the discrimination of lung cancer based on infrared spectroscopy. PLoS ONE.

[47] Wu Y., Chen K., Liu X., Huang L., Zhao D., Li L., Gao M., Pei D., Wang C., Liu X. (2015). Srebp-1 interacts with c-Myc to enhance somatic cell reprogramming. Stem Cells.

[48] Kumar D., Tiwari M., Goel P., Singh M.K., Selokar N.L., Palta P. (2024). Comparative transcriptome profile of embryos at different developmental stages derived from somatic cell nuclear transfer (SCNT) and in-vitro fertilization (IVF) in riverine buffalo (*Bubalus bubalis*). Vet. Res. Commun..

[49] Chen H., Zhang L., Guo Z., Wang Y., He R., Qin Y., Quan F., Zhang Y. (2015). Improving the development of early bovine somatic-cell nuclear transfer embryos by treating adult donor cells with vitamin C. Mol. Reprod. Dev..

[50] Qin H., Zhao A., Fu X. (2017). Small molecules for reprogramming and transdifferentiation. Cell. Mol. Life Sci..

[51] Rehman A., Fatima I., Noor F., Qasim M., Wang P., Jia J., Alshabrmi F.M., Liao M. (2024). Role of small molecules as drug candidates for reprogramming somatic cells into induced pluripotent stem cells: A comprehensive review. Comput. Biol. Med..

[52] Kim G., Roy P.K., Fang X., Hassan B.M., Cho J. (2019). Improved preimplantation development of porcine somatic cell nuclear transfer embryos by caffeine treatment. J. Vet. Sci..

[53] Sia J., Sun R., Chu J., Li S. (2016). Dynamic culture improves cell reprogramming efficiency. Biomaterials.

[54] Hawkins K.E., Joy S., Delhove J.M., Kotiadis V.N., Fernandez E., Fitzpatrick L.M., Whiteford J.R., King P.J., Bolanos J.P., Duchen M.R. (2016). NRF2 orchestrates the metabolic shift during induced pluripotent stem cell reprogramming. Cell Rep..

[55] Song Z., Cong P., Ji Q., Chen L., Nie Y., Zhao H., He Z., Chen Y. (2015). Establishment, differentiation, electroporation and nuclear transfer of porcine mesenchymal stem cells. Reprod. Domest. Anim..

[56] Secher J.O., Liu Y., Petkov S., Luo Y., Li D., Hall V.J., Schmidt M., Callesen H., Bentzon J.F., Sørensen C.B. (2017). Evaluation of porcine stem cell competence for somatic cell nuclear transfer and production of cloned animals. Anim. Reprod. Sci..

[57] Hu L., Lu Y., Xu H., Yang X., Lu S., Lu K. (2015). Production of hGFAP-DsRed transgenic Guangxi Bama mini-pigs via somatic cell nuclear transfer. Genet. Mol. Res..

[58] Cao J., Dong Y., Li Z., Wang S., Wu Z., Zheng E., Li Z. (2024). Treatment of donor cells with oxidative phosphorylation inhibitor CPI enhances porcine cloned embryo development. Animals.

[59] Zhang Y.T., Yao W., Chai M.J., Liu W.J., Liu Y., Liu Z.H., Weng X.G. (2022). Evaluation of porcine urine-derived cells as nuclei donor for somatic cell nuclear transfer. J. Vet. Sci..

[60] Hua Z.D., Guo S., Xiao H.W., Ren H.Y., Zhang L.P., Ge Y.W. (2017). Effect of oocytes in vivo and in vitro on the developmental potential of porcine somatic cell cloned embryos. Chin. Anim. Husb. Vet. Med..

[61] Hyun S., Lee G., Kim D., Kim H., Lee S., Kim S., Lee E., Lim J., Kang S., Lee B. (2003). Effect of maturation media and oocytes derived from sows or gilts on the development of cloned pig embryos. Theriogenology.

[62] Yuan Y., Spate S.L., Redel B.K., Tian Y.C., Zhou J., Prather R.S., Roberts R.M. (2017). Quadrupling efficiency in production of genetically modified pigs through improved oocyte maturation. Proc. Natl. Acad. Sci. USA.

[63] Zhao H.X., Xie S.Y., Zhang N., Ao Z., Wu X., Yang L.S., Shi J.S., Mai R.B., Zheng E.Q., Cai G.Y. (2020). Source and follicular fluid treatment during the in vitro maturation of recipient oocytes affects the development of cloned pig embryo. Cell. Reprogram..

[64] Shi J., Zhou R., Luo L., Mai R., Zeng H., He X., Liu D., Zeng F., Cai G., Ji H. (2015). Influence of embryo handling and transfer method on pig cloning efficiency. Anim. Reprod. Sci..

[65] Mordhorst B.R., Benne J.A., Cecil R.F., Whitworth K.M., Samuel M.S., Spate L.D., Murphy C.N., Wells K.D., Green J.A., Prather R.S. (2019). Improvement of in vitro and early in utero porcine clone development after somatic donor cells are cultured under hypoxia. Mol. Reprod. Dev..

[66] Ferraz M.A.M.M., Henning H.H.W., Costa P.F., Malda J., Melchels F.P., Wubbolts R., Stout T.A.E., Vos P.L.A.M., Gadella B.M. (2017). Improved bovine embryo production in an oviduct-on-a-chip system: Prevention of polyspermic fertilization and parthenogenic activation. Lab Chip.

[67] Moura M.T., Sousa R.V., Lucci C.M., Rumpf R. (2019). Bovine somatic cell nuclear transfer using mitomycin C-mediated chemical oocyte enucleation. Zygote.

[68] Wang X.Y., Qu J.D., Li J., He H.B., Liu Z.H., Huan Y.J. (2020). Epigenetic reprogramming during somatic cell nuclear transfer: Recent progress and future directions. Front. Genet..

[69] Gao L., Wu K.L., Liu Z.B., Yao X.L., Yuan S.L. (2018). Chromatin accessibility landscape in human early embryos and its association with evolution. Cell.

[70] Lu F.L., Liu Y.T., Inoue A., Suzuki T., Zhao K.J., Zhang Y. (2016). Establishing chromatin regulatory landscape during mouse preimplantation development. Cell.

[71] Pérez-Palacios R., Bourc’his D. (2018). A single-cell chromatin map of human embryos. Nat. Cell Biol..

[72] Wu J.Y., Huang B., Chen H., Yin Q.Z., Liu Y., Xiang Y.L., Zhang B.J., Liu B.F., Wang Q.J., Xia W.K. (2016). The landscape of accessible chromatin in mammalian preimplantation embryos. Nature.

[73] Nagano T., Lubling Y., Stevens T.J., Schoenfelder S., Yaffe E., Dean W., Laue E.D., Tanay A., Fraser P. (2013). Single-cell Hi-C reveals cell-to-cell variability in chromosome structure. Nature.

[74] Hochedlinger K., Jaenisch R. (2015). Induced pluripotency and epigenetic reprogramming. Cold Spring Harb. Perspect. Biol..

[75] Douglas C.M.W., Stemerding D. (2015). Challenges for the European governance of synthetic biology for human health. Life Sci. Soc. Policy.

